# Infection and treatment method (ITM) vaccine against East Coast fever: reducing the number of doses per straw for use in smallholder dairy herds by thawing, diluting and refreezing already packaged vaccine

**DOI:** 10.1186/s12917-019-1787-y

**Published:** 2019-01-31

**Authors:** Ekta Patel, Stephen Mwaura, Giuseppe Di Giulio, Elizabeth A. J. Cook, Godelieve Lynen, Philip Toye

**Affiliations:** 1 International Livestock Research Institute, P.O.Box 30709-00100, Old Naivasha Road, Nairobi, Kenya; 2 Veterinary Services Professionals Ltd, P.O.Box 13188, Arusha, Tanzania

**Keywords:** Infection and treatment method, Vaccine, *Theileria parva*, Smallholder, East Coast fever, Smallholder farmers

## Abstract

**Background:**

The Infection and Treatment Method (ITM) of vaccination is the only immunization procedure currently available to protect cattle against East Coast fever (ECF), a tick-transmitted disease responsible for losses of several hundreds of millions of dollars per year in sub-Saharan Africa. The vaccine comprises a homogenized preparation of infected ticks packaged in straws and stored in liquid nitrogen. The current manufacturing protocol results in straws containing 30–40 doses (ILRI 0804), which is impractical for immunizing small herds as found in dairy and smallholder farming systems. The ILRI 0804 SD stabilate was prepared as a 1:5 dilution of the parent stabilate, with the aim of producing vaccine stabilate straws containing between four to eight doses and thus suitable for smallholder farming systems. Infectivity of the diluted stabilate was assessed and the protective efficacy of the diluted stabilate was determined by performing experimental and field immunizations.

**Results:**

Two groups of six cattle were inoculated with 1 ml of the diluted stabilate at 1:20 (equivalent to the recommended field dose for ILRI 0804, assuming no loss of sporozoite viability during thawing and refreezing) and 1:14 (assuming 30–35% loss of sporozoite viability). Schizonts were detected in all 12 animals, showing viability of sporozoites. Ten animals from the infectivity study and two control animals not previously exposed to *T. parva* were challenged with the parental ILRI 0804 stabilate. The results show that the two control animals displayed severe ECF reactions and were treated 14 days after challenge. Of the previously infected animals, only one underwent a severe reaction following challenge, a result in accord with the challenge experiments performed previously with the parent stabilate [Ticks Tick-Borne Dis 7:306-314, 2016]. The animal that displayed a severe reaction had no detectable schizonts and did not seroconvert following the initial inoculation with ILRI 0804 SD. In addition, 62 animals immunized under field conditions showed a mean seroconversion rate of 82%.

**Conclusion:**

The results presented in this article demonstrate that it is possible to prepare straws suitable for use in smallholder herds by thawing, diluting and refreezing already packaged vaccine.

## Background

East Coast fever (ECF) is an economically important disease of cattle in both smallholder and pastoralist systems in 11 countries in sub-Saharan Africa. It is estimated that the disease is responsible for economic losses of over US$300 million per year [7, 10], with at least 40 million cattle in the region exposed to the disease [8]. ECF is caused by the tick-transmitted apicomplexan parasite, *Theileria parva,* and is characterised by a severe lymphoproliferative disorder. Mortality and morbidity rates in susceptible animals can approach 90% [2].

The only currently available method of immunization against ECF is known as the Infection and Treatment Method (ITM). The vaccine consists of a homogenized and partially purified preparation of infected ticks, which is administered simultaneously with long-acting oxytetacycline. The oxytetracycline serves to prevent the development of severe clinical disease from the otherwise lethal dose of sporozoites. Lynen et al. [5] demonstrated that ITM is the most effective method for ECF control in Tanzania’s smallholder dairy sector. The vaccine is expensive, costing at least US$7 per animal, although this is considerably less than the cost of treatment of US$40 per animal [5].

The most commonly used version of the ITM vaccine is the Muguga cocktail, which is a mixture of stabilates from three different *T. parva* isolates [14] and comprises multiple parasite types as demonstrated by genomic analysis of mini- and micro-satellites and polymorphic antigens [1, 12]. The recent commercial-scale productions of the ITM Muguga cocktail vaccine resulted in vaccine stabilate packaged at between 30 and 40 doses per 0.5 ml straw [13]. The straws are stored in liquid nitrogen to maintain sporozoite viability. These factors present practical difficulties for some livestock owners, such as smallholder farmers with small numbers of cattle to be vaccinated.

One potential approach to overcome this issue is to thaw, dilute, repackage and refreeze the stabilate from already dispensed straws. It does, however, rely on there being minimal loss of sporozoite viability or of key antigenic components of the vaccine during the repackaging process. In a previous study, Mbao et al. [6] showed that sporozoites from a single stabilate showed 33–35% loss of in vitro infectivity following thawing and refreezing. However, no dilution step or in vivo analysis was reported.

This paper describes a method for thawing, diluting, repackaging and refreezing the ITM Muguga cocktail vaccine stabilate to retain sporozoite infectivity and antigenicity, as judged by seroconversion and protection from challenge with the parent stabilate. The paper also presents results from the field immunizations where the newly diluted vaccine was tested and shown to be immunogenic, with seroconversion detectable in 82% of vaccinated animals.

## Results

The parent ILRI 0804 stabilate is recommended for use at a dilution of 1:100, which yields about 40 doses per straw. The diluted and repackaged stabilate described here (ILRI 0804 SD stabilate) was prepared as a 1:5 dilution of the parent stabilate, with the aim of producing vaccine stabilate straws containing between four to eight doses and thus suitable for smallholder farming systems.

### Determining the infectivity of the ILRI 0804 SD vaccine stabilate

In order to assess the infectivity of the diluted stabilate two groups of six cattle were inoculated with 1 ml of the stabilate, diluted either 1:20 or 1:14. The dilution of 1:20 represents a dose equivalent to the recommended field dose of ILRI 0804 (1:100) assuming no loss of viability, whereas the 1:14 dilution assumes there was about a 33–35% loss of infective sporozoites during repackaging [6]. The cattle were inoculated without simultaneous administration of oxytetracycline, in line with previous procedures [13]. The results (Table 1) showed that schizonts were detected in nine of the 12 animals, demonstrating that the stabilate contained viable, infective sporozoites. This was supported by the results of the serological testing, which showed that all but one of the animals, including two of those in which schizonts were not detected, seroconverted within 28 days of inoculation. There was no apparent correlation of the clinical outcome with dose, as each of the dilution groups had two animals showing moderate reactions. Similarly, two of the three animals in which there was no apparent reaction had received the 1:14 stabilate dilution.

**Table 1:** Clinical and parasitological responses in cattle inoculated with the ILRI 0804 SD vaccine stabilate at a dilution of 1:14 or 1:20

Animal	Days to fever	Duration of fever (d)	Days to schizonts	Duration of schizonts (d)	Days to piroplasms	ECF reaction	Day of seroconv.
Group 1. Immunizing Dose - 1:14							
BF 051	nd^a^	nd	12	2	nd	mild	21
BF 053	14	6	12	8	15	mod.^b^	21
BF 056	nd	nd	12	2	nd	mild	21
BF 061	nd	nd	nd	nd	nd	nr^c^	nd
BF 066	nd	nd	nd	nd	nd	nr	28
BF 069	13	1	10	5	15	mod.	21
Group 2. Immunizing Dose - 1:20							
BF 049	nd	nd	8	1	nd	mild	21
BF 052	nd	nd	nd	nd	nd	nr	28
BF 055	13	4	9	13	15	mod.	21
BF 060	11	7	9	6	14	mild	21
BF 065	nd	nd	11	4	nd	mild	21
BF 068	14	8	9	9	15	mod.	21

^a^nd = not detected

^b^mod. = moderate

^c^nr = no apparent reaction

### Determining the protective efficacy of ILRI 0804 SD by stabilate challenge

To determine whether the thawing and refreezing process resulted in loss of minor key antigenic components, the infected animals were challenged with the parent stabilate, in the expectation that any loss of major antigenic types would result in parasite breakthrough. This approach would also confirm if the animals had received a protective dose of sporozoites. Ten animals had survived untreated from the infectivity experiment, as one animal (BF053) died and another (BF055) was euthanized 36 and 24 days after inoculation, respectively, due to causes unrelated to ECF. All 10 animals and two control animals not previously exposed to *T. parva* were challenged with the parental ILRI 0804 stabilate.

The results (Table 2) show that the two control animals displayed severe ECF reactions and were treated 14 days after challenge. Of the previously infected animals, only one underwent a severe reaction following challenge, a result in accord with the challenge experiments performed previously with the parent stabilate [13]. The animal that displayed a severe reaction (BF061) had no detectable schizonts and did not seroconvert following the initial inoculation with ILRI 0804 SD. It would appear that this animal received a sub-optimal dose of viable sporozoites, although the reasons for this are not clear. In comparing the clinical outcomes of the two groups, it was noted that four of the animals in each group underwent mild reactions, indicating that there was very little difference in the protection afforded by the two dilutions. We were also able to compare difference in the mean time to and mean duration of parasitosis between the two groups. The analysis showed there was no statistically significant difference (*p* = 0.115–0.147 and 0.685–0.736, respectively).

**Table 2:** Clinical and parasitological responses in cattle challenged with ILRI 0804 vaccine stabilate

Animal	Days to fever	Duration of fever (d)	Days to schizonts	Duration of schizonts (d)	Days to piroplasm	ECF reaction
Group 1 Immunizing Dose 1:14						
BF 051	12	2	8	8	nd^a^	mild
BF 056	nd	nd	9	1	nd	mild
BF 061	11	10	11	8	15	severe
BF 066	nd	nd	8	2	nd	mild
BF 069	nd	nd	8	1	nd	mild
Group 2 Immunizing Dose 1:20						
BF 049	nd	nd	13	3	nd	mild
BF 052	8	7	8	10	15	mod^b^.
BF 060	nd	nd	11	5	nd	mild
BF 065	nd	nd	nd	nd	nd	mild
BF 068	nd	nd	13	2	nd	mild
Control						
BF 045	9	7	8	10	13	severe
BF 071	8	10	8	11	13	severe

^a^nd = not detected

^b^mod. = moderate

### Performance of the ILRI 0804 SD vaccine stabilate in a field immunization in Tanzania

Field immunizations were undertaken in various locations in northern Tanzania to assess the performance of the ILRI 0804 SD stabilate under field conditions. The stabilate was administered at a dilution (1:10), which would yield approximately four doses per straw and thus be suitable for small herds. The stabilate was administered simultaneously with oxytetracycline to mimic the expected field use of the vaccine.

Of the 63 calves included in the analysis, only two showed clinical signs after vaccination. One calf died 26 days after immunization from complications not necessarily related to ECF, while a second calf was treated with buparvaquone (Butalex, Coopers Animal Health, Nairobi) when it showed inappetence 17 days after immunization, and recovered immediately. The seroconversion rates following immunization varied from 75 to 87% among farms, with an overall mean rate of 82%. The results indicate that the ILRI 0804 SD stabilate is safe and immunogenic when used at 1:10 (four doses per straw). As no control (unvaccinated) groups were included in this study, it is not possible to conclude definitively that the vaccine was protective but two ECF cases were recorded in non-vaccinated animals on one farm during the observation period.

## Discussion

The current ITM Muguga Cocktail (ILRI 0804) is packaged and stored as 30–40 doses which is impractical for smallholder farmers with fewer cattle. Therefore, the ILRI 0804 SD stabilate was prepared as a 1:5 dilution of the parent stabilate after thawing and refreezing already packaged straws, with the aim of producing vaccine stabilate straws containing between four to eight doses and thus suitable for smallholder farming systems. The possibility that the thawing and refreezing process during the preparation of diluted stabilate results in unacceptable loss of sporozoite viability was addressed in an infectivity assay, with the results showing that the stabilate was sufficiently viable to induce parasitosis and antibody responses when administered at a practically useful dilution. The subsequent challenge experiment involving a lethal inoculation of the parent stabilate demonstrated that the ILRI 0804 SD stabilate was protective and that there was no observable loss in key antigenic components from the Muguga cocktail stabilate. The results of the field immunization reinforced these results, with high seroconversion rates and minimal clinical reactions after immunization. If used as in the field trial at a dilution of 1:10, the resultant pack size of four doses per straw is immensely attractive for use in the smallholder dairy sector. The resources required for close monitoring and clinical follow-up of the animals in the field for an extended period were beyond the scope of the current study and no definitive conclusions regarding field protection could be drawn. However, the observation that over 1 million doses of the ILRI 0804 batch of vaccine have been used with no confirmed reports of vaccine failure when administered correctly suggests that the ILRI 0804 SD stabilate will be safe and effective under field conditions when used at a dilution which should be commercially attractive in the field.

An alternative approach to producing a vaccine stabilate at 5–10 doses per straw is to prepare a more dilute stabilate for initial packaging. This has the disadvantages of requiring a greater liquid nitrogen storage capacity, a reliable estimate of how much of the bulk vaccine stabilate should be packaged at the more dilute preparation and accurate prediction of the potency of each vaccine batch. Although the approach described here may result in some loss of sporozoite viability, it does allow the preparation of diluted straws according to demand and reduction in the amount of space required for storage in liquid nitrogen. A further improvement to this approach would be to store the vaccine stabilate as a bulk preparation [11] and prepare appropriately diluted stabilate straws when needed.

## Conclusion

In summary, we have shown that the thawing, diluting and re-freezing of an already dispensed preparation of the ITM Muguga cocktail vaccine stabilate results in a viable preparation useful for smallholder herds. Although there will be slightly increased costs associated with the production of the diluted and repackaged stabilate, this should be offset by decreased wastage of thawed but unused vaccine associated with use of the 40-dose straws and a perceived willingness of smallholder farmers to pay more to protect highly valuable animals.

## Methods

### Animals

All animal procedures used in the infectivity and challenge trials were approved by ILRI’s Institute Animal Care and Use Committee (IACUC File numbers 2011.02 and 2011.06). The animals used in this study were *Bos indicus* (Boran) cattle aged between from 9 and 12 months and were brought from the ILRI Kapiti farm where they were maintained under a strict acaricide regime. Before use, all cattle were screened by enzyme-linked immunosorbent assay (ELISA) to detect prior exposure to *T. parva*, *Theileria mutans*, *Babesia bigemina*, and *Anaplasma marginale* [3, 4, 9, 15] and Bovine Leukosis Virus (IDEXX Leukosis Serum × 2 Ab Test, IDEXX, Westbrook, Me, USA). Giemsa-stained blood smears were examined for haemoparasites and *Ehrlichia* spp. Only animals that were negative in all assays were included in the experiments.

### Vaccine stabilate

The Muguga cocktail vaccine stabilate ILRI 0804 has been previously described [13]. One hundred, 0.5 ml straws of the stabilate were selected at random and used to produce the diluted and repackaged straws.

### Thawing, diluting and repackaging stabilates

The straws were placed on a steel tray floating in a 37 °C water bath for 2–3 min. The thawed straws were pierced with a fine needle at the sealed end to allow the stabilate to flow into a flask on ice with stirring. A total stabilate volume of 45 ml was recovered. To achieve a 1:5 dilution, 180 ml of cryoprotectant medium (Eagle’s minimal essential medium, with 3.5% bovine serum albumin and 7.5% glycerol) was added slowly for 5 min at 1 drop per second, followed by a gradual increase in the flow rate to 2 drops per second for 5 min. The remaining medium was added as a slow trickle. The newly diluted stabilate, named ILRI 0804 SD, was mixed for a further 5 min before being dispensed using a vacuum manifold into 0.5 ml straws sealed at one end and labelled ECF MC ILRI 0804 SD. The entire procedure from thawing to filling was completed in 65 min. The newly filled straws were placed into plastic goblets on ice and left at − 80 °C overnight. The following day they were transferred into liquid nitrogen for long-term storage.

### Infectivity trial of the ILRI 0804 SD stabilate

The viability of the diluted and refrozen stabilate was determined in an infectivity trial using 12 animals placed randomly into two groups of six. The groups received 1 ml of either a 1:14 or a 1:20 dilution of the ILRI 0804 SD, delivered by subcutaneous inoculation below and anterior to the right parotid lymph node (without oxytetracycline). The animals were monitored for clinical and parasitological reactions as described previously [13]. Seroconversion to *T. parva* was assessed with the indirect ELISA [4] on sera collected at seven-day intervals from the day of inoculation. A percent positivity value of 19 or greater was considered positive.

### Challenge trial

Fifty days after inoculation, the surviving animals from the infectivity trial were challenged with a lethal dose of the parent stabilate ILRI 0804 (1 ml undiluted) administered as above. Two control animals not previously exposed to *T. parva* were included to ensure that the challenge dose and procedure was appropriate. The animals were monitored for clinical and parasitological reactions as before.

### Field immunizations

Field immunizations were undertaken in seven locations in northern Tanzania, six smallholder dairy farms in peri-urban Arusha/Arumeru District and one commercial Boran farm, between November 2011 and November 2012. The calves at the smallholder dairy farms were mainly *Bos taurus* (Jersey or Friesian) aged 1–4 months and the commercial farm had *Bos indicus* Boran calves, aged 1–4 months. The animals were immunized by inoculation with 1 ml of a 1:10 dilution of the ILRI 0804 SD stabilate as above, together with 30% long acting oxytetracycline administered intramuscularly. The animals were monitored for indications of ECF following vaccination. Serum samples were collected from the day of immunization and between 32 and 50 days following immunization and were assessed for anti-*T. parva* antibodies as above. Following removal of some samples due to pre-immunization positivity, the sera from 63 cattle were analysed.

### Statistical analysis

Differences in clinical outcomes (time to and duration of parasitosis) between the groups in the challenge trial were assessed using a random permutation t-test equivalent run 10 times to establish a range in the R software version 3.0.2 (R Core Team, 2013).

## Abbreviations

ECF: East Coast fever
ILRI: International Livestock Research Institute
ITM: Infection and treatment method
